# The diagnostic and prognostic value of serum human kallikrein-related peptidases 11 in non-small cell lung cancer

**DOI:** 10.1007/s13277-014-1674-x

**Published:** 2014-02-09

**Authors:** Chun-Hua Xu, Yu Zhang, Li-Ke Yu

**Affiliations:** 1 Department of Respiratory Medicine, Nanjing Chest Hospital, 215 Guangzhou Road, Nanjing, 210029 China; 2 Nanjing Clinical Center of Respiratory Diseases, Nanjing, China

**Keywords:** Kallikrein-related peptidases 11, Non-small cell lung cancer, Diagnosis, Prognosis

## Abstract

The aim of this study was to explore the diagnostic and prognostic value of serum human kallikrein-related peptidases 11 (KLK11) level in non-small cell lung cancer (NSCLC). Serum specimens from 138 patients with NSCLC and 40 healthy controls were collected. The concentration of KLK11 was measured by enzyme-linked immunosorbent assay (ELISA). The concentration of KLK11 in NSCLC was significantly higher compared to that in the controls (*P* < 0.01). The serum KLK11 levels decreased with stage, presence of lymph node, and distant metastases, regardless of histology, age, and sex. With a cutoff point of 1.05 ng/ml, KLK11 showed a good diagnostic performance for NSCLC. Univariate analysis revealed that NSCLC patients with serum high KLK11 had a longer overall survival (OS) and progression-free survival (PFS) than those with low KLK11 (HR of 0.36, *P* = 0.002; HR of 0.46, *P* = 0.009). Cox multivariate analysis indicated that KLK11 was an independent prognostic indicator of PFS and OS (HR of 0.53, *P* = 0.042; HR of 0.48, *P* = 0.037). Kaplan–Meier survival curves further confirmed that patients with high KLK11 have longer PFS and OS (*P* = 0.003 and *P* = 0.018, respectively). In conclusion, the measurement of KLK11 might be a useful diagnostic and prognostic test for NSCLC patients.

## Introduction

Lung cancer is the leading cause of cancer-related death worldwide, with more than 1.2 million deaths each year [1]. Non-small cell lung cancer (NSCLC) accounts for 80–85 % of total lung malignancies [2]. Although advances in noninvasive methods have improved our ability to detect lung cancer, more than 75 % of lung cancer patients present an advanced stage of disease [3], and they have little prospect of effective and curative treatment, with 5-year survival rates of less than 15 % [4].

Tumor markers play a key role in patient management for many malignancies. The potential uses of serum tumor markers include aiding early diagnosis, determining prognosis, prospectively predicting response or resistance to specific therapies, and monitoring therapy in patients with advanced disease. Kallikrein-related peptidases 11 (KLK11) is a member of the human kallikrein gene family, which localized on chromosome 19q13.4 [5]. Recent studies have reported that KLK11 has been expressed in many cancers, including prostate cancer [6], ovarian cancer [7], gastric cancer [8], as well as rectal carcinoma [9]. An immunofluorometric assay study demonstrated that KLK11 expression in ovarian cancer tissues is a marker of favorable prognosis, since patients with KLK-positive tumors exhibit a longer progression-free survival (PFS) and overall survival (OS) [10]. Additionally, Sasaki et al. [11] reported that lower KLK11 mRNA expression in lung cancer is an indicator of poor prognosis in patients with lung cancer. However, there seems to be a paucity of research concerned with serum KLK11 expression in NSCLC. For this reason, the goal of the present study was to investigate the baseline serum levels of KLK11 in patients with NSCLC to determine its potential diagnostic and prognostic roles.

## Materials and methods

### Patients

A total of 138 patients with NSCLC were examined at the Nanjing Chest Hospital between January 2006 and May 2008. The cohort of patients included 80 (58.0 %) male and 58 (42.0 %) female subjects, with a median age of 56 years (range 45–68 years). The clinical features of the patients are summarized in Table 1. Follow-up lasted through December 2012, with a median follow-up period of 22 months for living patients (range 3–80 months). PFS was defined as the time interval between the date of diagnosis and the date of disease relapse. OS was defined as the time interval between the date of diagnosis and the date of death.

**Table 1:** Clinical characteristics of NSCLC patients and controls

Variables	NSCLC	Control	*P* value
Subject, no.	138	40	
Age, year	57.8 ± 10.2	54.6 ± 7.8	0.614
Male/Female	80/58	26/14	0.325
Histology			
AC	78		
SCC	60		

*AC* adenocarcinoma, *SCC* squamous cell carcinoma

The diagnosis of lung cancer was made using various methods: sputum cytology, fine-needle aspiration or bronchoscopy, as dictated by the patient’s presentation. Pathologists interpreted the cytology or histology of tissue biopsy. Lung cancer was staged using a widely used classification system, and the staging procedure included a clinical examination; CT of the chest, abdomen, and brain; abdominal ultrasonography; bone scanning; and positron emission tomography.

The study protocol was approved by the ethics committee of Nanjing Chest Hospital. All patients provided written informed consent before enrollment.

### Measurement of serum KLK11 levels

Serum samples from each individual were obtained at the time of diagnosis, before any therapeutic measures were started (surgery, chemotherapy, or radiation). Samples were centrifuged at 1,500×*g* for 10 min at −4 °C. The supernatant was stored at −80 °C for assessment of the levels of KLK11. The KLK11 concentration was determined by ELISA with the commercial KLK11 ELISA Ready-SET-Go kit (eBioscience, San Diego, CA). All samples were blinded to the technologists running the assays, and the code was broken to the statisticians after the database was constructed.

### Statistical analysis

Statistical software (SPSS for Windows, version 18) was used for the analysis. Differences between independent groups were examined by the Mann–Whitney *U* test. To determine the diagnostic accuracy of KLK11, receiver operating characteristic (ROC) curves were retrieved from logistic regression analysis and the area under the curve (AUC) was calculated. Univariate survival analysis was performed using the Kaplan–Meier method and the log-rank test. Multivariate analysis was conducted to determine an independent impact on survival using the Cox proportional hazard method. *P* < 0.05 was considered statistically significant.

## Results

### Comparison of serum KLK11 levels between NSCLC patients and controls

**Fig. 1:**
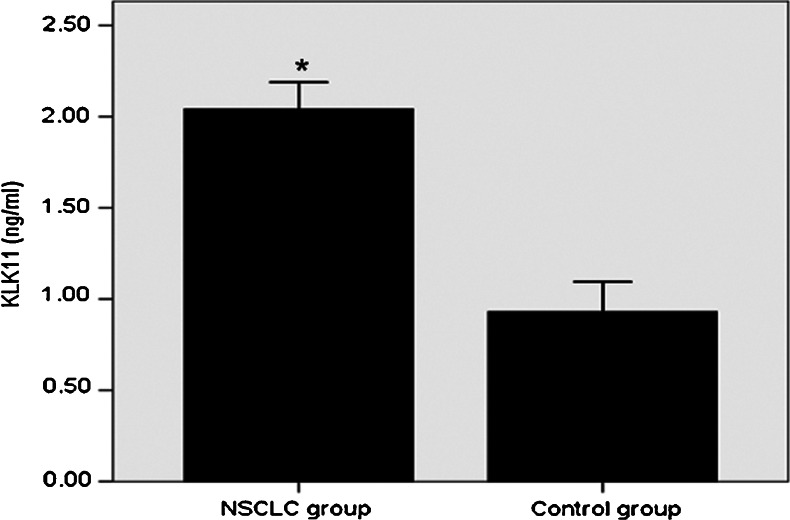
Levels of KLK11 in NSCLC. Among 138 NSCLC patients, the serum levels of KLK11 were 2.04 ± 0.86 ng/ml, which were significantly higher than 0.93 ± 0.52 ng/ml in healthy controls (*P* < 0.01)

As shown in Fig. 1, the concentration of KLK11 was significantly higher in patients with NSCLC (2.04 ± 0.86 ng/ml) than in those with the controls (0.93 ± 0.52 ng/ml) (*P* < 0.01).

### Diagnostic value of KLK11 in NSCLC

**Fig. 2:**
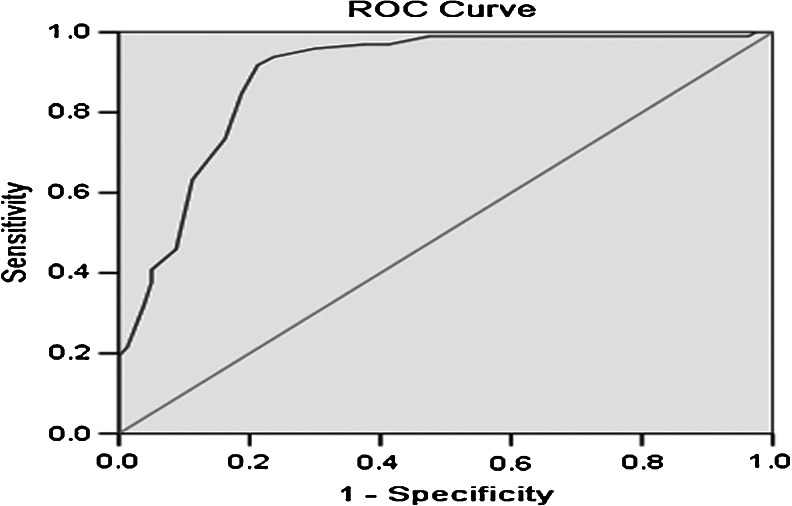
ROC of KLK11 for the diagnosis of NSCLC. Serum levels of KLK11 among 138 NSCLC patients and 40 healthy controls were determined. The diagnostic potentials of KLK11 were assessed by ROC curves. The AUC value was 0.892

A ROC curve analysis was carried out to assess the value of KLK11 in NSCLC. The area under the ROC curve was 0.892 (confidence interval (95 % CI) 0.841–0.942). With a cutoff point of 1.05 ng/ml, which was defined as the normal value based on the mean value, plus two standard deviation obtained from healthy controls, serum KLK11 has a sensitivity of 65.9 % (91/138), a specificity of 82.5 % (33/40), an accuracy of 69.7 % (124/178), a positive predictive value of 92.9 % (91/98), and a negative predictive value of 41.3 % (33/80) (Fig. 2).

### Relationship between serum KLK11 levels and clinicopathologic factors

The relationships between KLK11 levels and clinicopathologic factors of lung cancer patients are shown in Table 2. The serum KLK11 levels did not differ significantly with age (*P* = 0.569), sex (*P* = 0.505), or histology (*P* = 0.713). The levels of KLK11 were significantly correlated with tumor-node-metastasis (TNM) stage (*P* = 0.000), lymph node metastases (*P* = 0.000), and distant metastases (*P* = 0.000).

**Table 2:** The clinicopathological factors of NSCLC and the association with KLK11 levels

Factors	n	KLk11 (ng/ml)	*P*- value
Age, year			0.569
≥60	62	2.07 ± 0.77	
<60	76	2.12 ± 0.66	
Gender			0.505
Male	80	2.16 ± 0.82	
Female	58	1.99 ± 0.53	
Histology			0.713
AC	78	2.05 ± 0.85	
SCC	60	2.01 ± 0.53	
TNM stage			0.000
I–II	88	2.51 ± 0.61	
III–IV	50	1.76 ± 0.63	
Lymph node metastases			0.000
Absent	68	2.41 ± 0.64	
Present	70	1.65 ± 0.57	
Distant metastases			0.000
Absent	98	2.38 ± 0.59	
Present	40	1.89 ± 0.71	

*AC* adenocarcinoma, *SCC* squamous cell carcinoma

### Association of serum KLK11 levels with survival

Finally, we determined whether the baseline serum concentration of KLK11 would be a prognostic marker in NSCLC. The cutoff point of 1.05 ng/ml was selected to categorize patients as KLK11-high or low. Univariate analysis showed that serum KLK11 level was significantly correlated OS (*P* = 0.002) and PFS (*P* = 0.009) (Table 3).

**Table 3:** Univariate and multivariate analysis of KLK11 status with regard to PFS and OS

Variables	PFS			OS		
	HR	95 % CI	*P* value	HR	95 % CI	*P* value
Univariate analysis						
KLK11 (Low vs. High)	0.46	0.25–0.82	0.009	0.36	0.19–0.69	0.002
Age (≥60 vs. <60)	1.23	0.67–2.28	0.506	1.18	0.59–2.13	0.792
Gender (Male vs. Female)	1.32	0.71–1.82	0.782	1.19	0.69–1.98	0.673
Histology (AC vs. SCC)	1.83	0.59–2.13	0.792	1.34	0.65–1.98	0.546
Stage (I–II vs. III–IV)	1.33	0.65–2.21	0.001	0.93	1.09–3.44	0.025
Lymph node metastases (absent vs. present)	1.42	1.04–1.94	0.271	1.77	0.32–1.66	0.347
Distant metastases (absent vs. present)	1.98	1.03–3.01	0.039	1.87	1.04–2.99	0.075
Multivariate analysis						
KLK11 (low vs. high)	0.53	0.29-0.97	0.042	0.48	0.24-0.95	0.037
Age (≥60 vs. <60)	0.98	0.52-1.94	0.834	1.06	1.28-3.01	0.128
Gender (male vs. Female)	1.28	0.67-1.89	0.672	1.14	0.46-2.14	0.542
Histology (AC vs. SCC)	1.37	1.04-2.33	0.315	1.26	0.64-2.56	0.424
Stage (I–II vs. III–IV)	1.25	0.56-2.26	0.001	1.96	1.02-3.77	0.043
Lymph node metastases (absent vs. present)	1.13	0.81-1.57	0.148	1.84	0.33-1.72	0.334
Distant metastases (absent vs. present)	1.44	0.85-1.97	0.098	1.89	0.99-2.35	0.051

*HR* hazard ratio, *CI* confidence interval

**Fig. 3:**
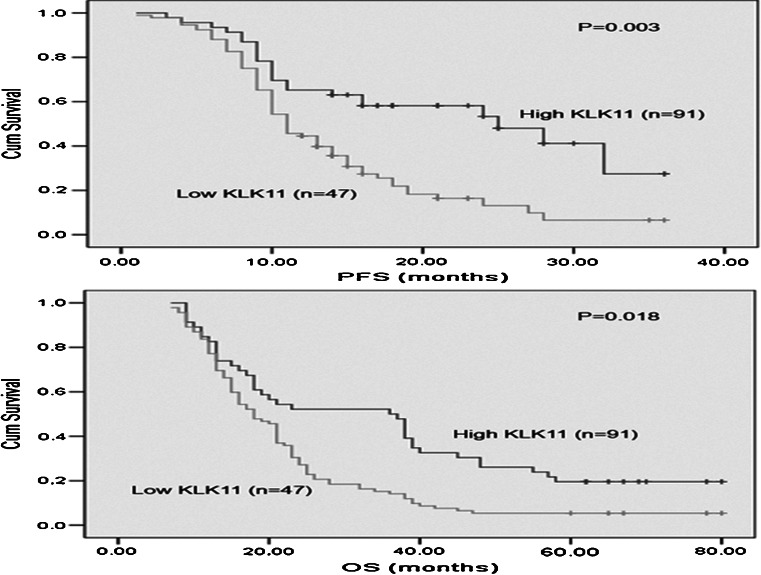
Kaplan–Meier survival curves for PFS and OS in patients with KLK11-high and -low NSCLC. Log-rank test determined that the PFS and OS in high KLK11 group were significantly longer than those in the low KLK11 group (*P* = 0.003; *P* = 0.018)

In multivariate analysis, high KLK11 was found to be significantly associated with a longer PFS and OS (HR 0.53 and 0.48; *P* = 0.042 and *P* = 0.037, respectively). Kaplan–Meier survival curves (Fig. 3) further demonstrate that lung cancer patients with high KLK11 have substantially longer PFS and OS (*P* < 0.05), compared to those with low KLK11 cancer. As expected, disease stage was found to be strongly associated with decreased PFS and OS, in both univariate and multivariate analyses (*P* < 0.05).

## Discussion

During the last few years, numerous studies have been published which attempt to refine our understanding of determinants of prognosis in lung cancer by analyzing tumor-associated markers thought to be of biologic relevance in the carcinogenic process. Proteolytic enzymes of several catalytic classes have emerged as important prognostic factors in cancer [12]. Among these enzymes are many members of human tissue kallikrein family of secreted serine proteases, including KLK11, a promising biomarker for lung cancer diagnosis, and prognosis [11,13].

In the present study, serum KLK11 levels were significantly elevated in patients with lung cancer compared with control subjects, making them potential adjunctive tools for diagnosis of lung cancer. Furthermore, at a cutoff point of 1.05 ng/ml, KLK11 had a sensitivity of 91.3 % and a specificity of 72.5 % for the prediction of lung cancer. Importantly, the serum KLK11 levels did not differ significantly with age, gender, and histology. The levels of KLK11 were significantly correlated with TNM stage, the presence of lymph node, and distant metastases.

Several previous studies have reported an association between kallikrein mRNA expression and cancer prognosis [14–16]. KLK5 and KLK4 have been associated with poor prognosis in ovarian cancer, and KLK5 has also been shown to be associated with poor prognosis in breast cancer [17,18]. In contrast, KLK8 and KLK9 expression have been reported to be favorable prognosis in ovarian cancer [19,20]. In addition, KLK12 is reported to be an independent and favorable prognostic marker for breast cancer [21]. Sasaki et al. [11] have indicated that there is a significant correlation between decreased KLK11 mRNA expression level and poor prognosis in lung cancer. This study supports the increasing body of literature demonstrating the expression of kallikrein family gene involvement in the prognosis of human cancers. The most striking association we observed in NSCLC patients was a significant correlation between increased KLK11 level and favorable prognosis. We have demonstrated that high KLK11 was significantly associated with an increased PFS and OS in univariate analysis. This relationship was further illustrated in the Kaplan–Meier survival curves. Multivariate analysis also indicated that KLK11 was an independent indicator of PFS and OS.

In conclusion, our data suggest that serum KLK11 may be a useful diagnostic biomarker and shows a promising potential as prognostic marker in NSCLC patients. More large-scale prospective studies are warranted to confirm the findings.

## References

[1] Chen Z, Wang T, Cai L, Su C, Zhong B, Lei Y (2012). Clinicopathological significance of non-small cell lung cancer with high prevalence of Oct-4 tumor cells. J Exp Clin Cancer Res.

[2] Smith RA, Cokkinides V, Brawley OW (2009). Cancer screening in the United States, 2009: a review of current American Cancer Society guidelines and issues in cancer screening. CA Cancer J Clin.

[3] Oguz A, Unal D, Tasdemir A, Karahan S, Aykas F, Mutlu H (2013). Lack of any association between blood groups and lung cancer, independent of histology. Asian Pac J Cancer Prev..

[4] Jemal A, Siegel R, Xu J, Ward E (2010). Cancer statistics, 2010. CA Cancer J Clin.

[5] Sano A, Sangai T, Maeda H, Nakamura M, Hasebe T, Ochiai A (2007). Kallikrein 11 expressed in human breast cancer cells releases insulin-like growth factor through degradation of IGFBP-3. Int J Oncol.

[6] Luo LY, Shan SJ, Elliott MB, Soosaipillai A, Diamandis EP (2006). Purification and characterization of human Kallikrein 11, a candidate prostate and ovarian cancer biomarker, from seminal plasma. Clin Cancer Res.

[7] McIntosh MW, Liu Y, Drescher C, Urban N, Diamandis EP (2007). Validation and characterization of human Kallikrein-11 as a serum marker for diagnosis of ovarian carcinoma. Clin Cancer Res.

[8] Unal D, Tasdemir A, Oguz A, Eroglu C, Cihan YB, Turak EE (2013). Is human Kallikrein-11 in gastric cancer treated with surgery and adjuvant chemoradiotherapy associated with survival?. Pathol Res Pract.

[9] Yu X, Tang HY, Li XR, He XW, Xiang KM (2010). Overexpression of human kallikrein 11 is associated with poor prognosis in patients with low rectal carcinoma. Med Oncol.

[10] Diamandis EP, Borgoño CA, Scorilas A, Harbeck N, Dorn J, Schmitt M (2004). Human kallikrein 11: an indicator of favorable prognosis in ovarian cancer patients. Clin Biochem.

[11] Sasaki H, Kawano O, Endo K, Suzuki E, Haneda H, Yukiue H (2006). Decreased Kallikrein 11 messenger RNA expression in lung cancer. Clin Lung Cancer.

[12] Lei KF, Liu BY, Zhang XQ, Jin XL, Guo Y, Ye M (2012). Development of a survival prediction model for gastric cancer using serine proteases and their inhibitors. Exp Ther Med.

[13] Planque C, Li L, Zheng Y, Soosaipillai A, Reckamp K, Chia D (2008). A multiparametric serum kallikrein panel for diagnosis of non-small cell lung carcinoma. Clin Cancer Res.

[14] Alexopoulou DK, Papadopoulos IN, Scorilas A (2013). Clinical significance of kallikrein-related peptidase (KLK10) mRNA expression in colorectal cancer. Clin Biochem.

[15] Talieri M, Alexopoulou DK, Scorilas A, Kypraios D, Arnogiannaki N, Devetzi M (2011). Expression analysis and clinical evaluation of kallikrein-related peptidase 10 (KLK10) in colorectal cancer. Tumour Biol.

[16] Patsis C, Yiotakis I, Scorilas A (2012). Diagnostic and prognostic significance of human kallikrein 11 (KLK11) mRNA expression levels in patients with laryngeal cancer. Clin Biochem.

[17] Xi Z, Kaern J, Davidson B, Klokk TI, Risberg B, Tropé C (2004). Kallikrein 4 is associated with paclitaxel resistance in ovarian cancer. Gynecol Oncol.

[18] Yousef GM, Scorilas A, Kyriakopoulou LG, Rendl L, Diamandis M, Ponzone R (2002). Human kallikrein gene 5 (KLK5) expression by quantitative PCR: an independent indicator of poor prognosis in breast cancer. Clin Chem.

[19] Kountourakis P, Psyrri A, Scorilas A, Markakis S, Kowalski D, Camp RL (2009). Expression and prognostic significance of kallikrein-related peptidase 8 protein levels in advanced ovarian cancer by using automated quantitative analysis. Thromb Haemost.

[20] Borgoño CA, Kishi T, Scorilas A, Harbeck N, Dorn J, Schmalfeldt B (2006). Human kallikrein 8 protein is a favorable prognostic marker in ovarian cancer. Clin Cancer Res.

[21] Talieri M, Devetzi M, Scorilas A, Pappa E, Tsapralis N, Missitzis I (2012). Human kallikrein-related peptidase 12 (KLK12) splice variants expression in breast cancer and their clinical impact. Tumour Biol.

